# AR Signaling and the PI3K Pathway in Prostate Cancer

**DOI:** 10.3390/cancers9040034

**Published:** 2017-04-15

**Authors:** Megan Crumbaker, Leila Khoja, Anthony M. Joshua

**Affiliations:** 1 Kinghorn Cancer Centre, St Vincent’s Hospital, 370 Victoria Street, Darlinghurst, Sydney, NSW 2010, Australia; m.crumbaker@garvan.org.au; 2 Garvan Institute of Medical Research, St Vincent’s Clinical School, University of New South Wales, Sydney, 384 Victoria St, Darlinghurst, Sydney, NSW 2010, Australia; 3 AstraZeneca UK, Clinical Discovery Unit, Early Clinical Development Innovative Medicines, da Vinci Building, Melbourn Science Park, Melbourn, Hertfordshire SG8 6HB, UK; lkhoja@yahoo.com; 4 Addenbrookes Hospital, Cambridge University Hospitals NHS Foundation Trust Cambridge Biomedical Campus, Hills Rd, Cambridge CB2 0QQ, UK; 5 Princess Margaret Cancer Centre, University Health Network, University of Toronto, University Avenue, Toronto, ON M5G 2M9, Canada

**Keywords:** PI3K, prostate cancer, AR signaling, castrate resistant prostate cancer

## Abstract

Prostate cancer is a leading cause of cancer-related death in men worldwide. Aberrant signaling in the androgen pathway is critical in the development and progression of prostate cancer. Despite ongoing reliance on androgen receptor (AR) signaling in castrate resistant disease, in addition to the development of potent androgen targeting drugs, patients invariably develop treatment resistance. Interactions between the AR and PI3K pathways may be a mechanism of treatment resistance and inhibitors of this pathway have been developed with variable success. Herein we outline the role of the PI3K pathway in prostate cancer and, in particular, its association with androgen receptor signaling in the pathogenesis and evolution of prostate cancer, as well as a review of the clinical utility of PI3K targeting.

## 1. Introduction 

Prostate cancer is the second most common non-cutaneous cancer in men and the fifth cause of cancer death in men worldwide [1]. The understanding that androgen receptor signaling continues to influence the evolution and development of metastatic castrate-resistant prostate cancer (mCRPC) has prompted the development of novel androgen pathway targeting agents such as enzalutamide and abiraterone acetate. These drugs have yielded practice-changing results with improvements in overall survival as well as a number of meaningful surrogate endpoints. Both enzalutamide and abiraterone are now licensed for the treatment of mCRPC pre- or post-chemotherapy [2,3,4,5].

However, resistance to these agents invariably develops via multi-factorial mechanisms [6,7]. It is generally believed that strategies to target inherent and or acquired resistance will lead to more efficacious therapeutic combinations. Activation of the phosphatidylinositol 3-kinase (PI3K) pathway is seen commonly in castrate-resistant disease, and this pathway may represent a therapeutic target with which to overcome treatment resistance. Herein we outline the role of the PI3K pathway in prostate cancer and, in particular, its association with androgen receptor signaling in the pathogenesis and evolution of prostate cancer as well as a review of the clinical utility of PI3K targeting.

## 2. The Androgen Receptor Pathway

The AR is a ligand-dependent nuclear transcription factor expressed in a variety of tissues which, in the absence of ligand, remains in the cytosol bound to heat shock proteins (Hsps). Though numerous ligands interact with the AR, its predominant native ligands are the androgens, 5α-dihydrotestosterone (DHT) and testosterone. The binding of these ligands to the AR initiates male sexual development and pubertal changes in addition to maintaining libido, spermatogenesis, muscle mass, erythropoiesis and bone mineral density in adult males [8].

Once the AR is engaged its effects manifest via three mechanisms. Firstly, classical AR signaling occurs when androgen binds to the ligand binding domain (LBD) to displace the Hsps triggering AR dimerization, phosphorylation and conformational change leading to exposure of the nuclear localization sequence (NLS). The AR then translocates to the nucleus and the DNA binding domain (DBD) binds to androgen responsive elements (AREs) to induce transcription of specific AR-responsive genes that recruit transcription co-activators and co-suppressors [9,10]. Alternatively, the androgen/AR complex can also trigger second messenger pathways leading to activation of several signaling cascades including MAPK/ERK and AKT [10,11]. This occurs in the cytosol through non-nuclear signaling and is rapid in onset as compared to classical signaling [10,12]. Thirdly ligand-independent activation of the AR is possible via growth factors (such as cytokines e.g., IL-6 [13,14]) and subsequent protein kinase and MAPK pathway activation, phosphorylation of the AR or co-activator stimulation such as insulin-like growth factor (IGF) activation of the AR [15,16]. Such alternative activation can stimulate distinct genes compared to classical AR signaling and may be particularly important in mCRPC [6].

In the normal prostate gland, AR is expressed in the stromal and epithelial compartments [12,17]; postnatal development of the gland is dependent on reciprocal signaling between these two compartments [18]. AR is expressed in both basal and luminal cells of the prostatic epithelium where its primary role is to promote expression of genes involved in terminal differentiation, secretion and suppression of proliferation to maintain homeostasis [12,19,20,21,22,23,24].

## 3. AR Signaling in Prostate Cancer

Aberrant AR signaling is critical to the evolution of prostatic carcinogenesis. The AR has been shown to be necessary for cell proliferation, survival and invasion in early and late prostate cancer [25,26,27]. Rates of cell proliferation and programmed cell death are balanced in the normal prostatic epithelium but this balance is lost in prostate cancer cells [28]. The mechanism for the switch from homeostatic to proliferative AR signaling in prostate cancer is unknown [12]. AR-regulated cancer-specific gene fusions are relatively common and may play a role. Fusion of the ARE-containing promoter from the AR target gene TMPRSS2 to the coding sequence of several members of the Ets family has been well-described [29,30]. These fusions result in AR-driven production of Ets transcription factors potentially leading to proliferation and promotion of cell survival. These fusions however are not present in all tumors. Alternatively, studies mapping genomic binding sites of the AR using ChIP technology have revealed that direct AR binding to aberrant targets may drive prostate pathogenesis [31].

The reliance of prostate cancer on AR signaling has led to the development of potent androgen pathway targeted treatments. Despite initial responses in many however, resistance to these agents is inevitable and remains an intractable problem. Resistance to these therapies may occur broadly through at least three mechanisms [6,10,12,15,24,32,33,34,35]: (1) AR-independent activation of AR-dependent pathways via bypass mechanisms, such as through up-regulation of glucocorticoid receptor expression [32]; (2) De-differentiation such as BRN2-mediated trans-differentiation to neuroendocrine prostate carcinoma [36]; and (3) The most commonly targeted mechanism, direct reactivation of the AR and its signaling despite castrate levels of androgens. The third mechanism can occur via AR gene amplification or AR protein overexpression. It may be ligand-dependent, such as intra-tumoral androgen synthesis activating classical signaling and AR LBD mutations leading to increased sensitivity to agonists or alternate non-androgen ligands [37,38]. Conversely, AR reactivation may also be ligand-independent; examples include AR splice variants resulting in constitutive activation [39,40,41,42] (reviewed by Sprenger and Plymate [43]) or AR activation through other proliferation pathways. The PI3K pathway may be involved in more than one of the above mechanisms through non-nuclear interactions between ligand-activated AR and PI3K [10,12] and direct stimulatory feedback from the PI3K pathway [44].

## 4. The PI3K Signaling Pathway

PI3Ks are a family of lipid kinases that regulate anabolic and catabolic activities in the cell through phosphorylation of the 3′-hydroxyl group of phosphoinositides and phosphatidylinositol. PI3Ks are divided into three classes according to their preferred substrate and sequence homology with class IA thought to be most relevant to human cancers [45].

Class IA PI3Ks are heterodimers made up of a regulatory subunit (p85α, p55α, p50α, p85β or p85γ) and a catalytic subunit (p110α, β or δ) that can be activated by receptor tyrosine kinases, G-protein coupled receptors or oncogenes [46,47]. Following stimulation, the catalytic subunit of PI3K phosphorylates phosphatidylinositol-4,5-biphosphate (PIP2) to phosphatidylinositol-3,4,5-triphosphate (PIP3), a reaction negatively regulated by the phosphatase and tensin homolog chromosome 10 (PTEN) and INPP4B. PIP3 acts as a second messenger to propagate intracellular signaling by binding pleckstrin homology domains. This signaling cascade eventually leads to AKT activation through phosphorylation by PDK1 and the mTORC 1/2 complexes. AKT, in turn, phosphorylates several cellular proteins which regulate cellular processes including cell growth, survival, proliferation, metabolism and angiogenesis through effectors such as p27, BAD, glycogen synthetase kinase 3 (GSK3) and forkhead box O (FOXO) transcription factors [46].

PIK3CA, PIK3CB and PIK3CD genes encode the p110α, β and δ isoforms respectively. The p110α and 110β isoforms are both widely expressed but p110δ is generally only found in leucocytes. Both the alpha and beta isoforms generate PIP3 but have differing roles: p110α is mainly found in the cytoplasm and is crucial in insulin signaling, glucose metabolism and G1 cell cycle entry while p110β is found in the nucleus and is important in DNA synthesis and replication and cell mitosis [48]. Both isoforms have been implicated in human cancer. Oncogenicity of the p110α isoform is well established [49,50,51,52] and mutations of PIK3CA play a causative role in the development of many cancer types (reviewed by Samuels [53]). PIK3CB and PIK3CD genes are rarely mutated in cancers but are often amplified or over-expressed [54]. Aberrant PI3K signaling in cancer can also occur via PTEN abnormalities including mutations, promotor hypermethylation or loss of heterozygosity; AKT isoform mutations or amplifications can occur as well (reviewed by Sadeghi and Gerber [55]).

## 5. PI3K Pathway Activation in Prostate Cancer

Aberrations in PI3K/AKT/mTOR signaling have been identified in approximately 40% of early prostate cancer cases and 70–100% in advanced disease [56,57]. In particular, loss of PTEN leading to constitutive activation of the PI3K pathway has been documented in 30% of primary and 60% of castrate-resistant prostate cancers [58]. Activation of the PI3K pathway is associated with resistance to androgen deprivation therapy, disease progression and poor outcomes in prostate cancer [59,60,61,62]. Over-activation via PTEN loss has been shown to initiate prostate cancer development. Varying rates of prostatic hyperplasia and cancer are seen in mouse models with heterozygous loss of PTEN [63,64,65,66] and combined deletion of a second tumor suppressor gene can induce prostate cancer with complete penetrance in some models [67]; heterozygous models failed to develop metastatic disease however. Conditional PTEN knockout mice though can mimic the course of human prostate cancer with progression from hyperplasia to invasive cancer to metastatic disease [68]. Moreover, pre-clinical data demonstrate that some PTEN-deficient neoplasms, including prostate cancer, particularly activate the PI3K pathway through the p110β isoform of the PI3K catalytic subunit [69,70,71]. Ablation of p110β but not p110α inhibits downstream AKT signaling resulting in reduced tumorogenesis in these models. Importantly however, selective p110β inhibition only temporarily inhibits signaling in PTEN deficient models because it removes feedback inhibition on receptors which in turn up-regulate signaling via p110α [72]. Combined inhibition of p110α and p110β results in more sustained suppression of signaling with improved tumor shrinkage in PTEN null models of prostate cancer as compared to p110β inhibition alone.

The association of PI3K pathway activation with castrate-refractory disease suggests that a critical component of the poor prognostic value of PI3K aberrations may be its interaction with androgen signaling. Additionally, responses to AR inhibitors in prostate cancers with PTEN loss may depend on the level of PI3K pathway activation.

## 6. Interaction of PI3K and AR Signaling

Despite the association outlined above, the effect of PI3K activation on prostate cancer growth pre-clinically is not dichotomous as some cell lines with PTEN loss (e.g., LNCaP) retain sensitivity to castration, while the robust response to castration in de novo disease suggests that most PTEN null tumors retain some sensitivity to androgen deprivation. The mechanism of the interaction between these two pathways remained unclear until relatively recently.

Two landmark papers defined the interplay between PTEN loss/PI3K activation and AR signaling in the development of prostate cancer [56,73]. Carver et al. first demonstrated in a series of studies on PTEN deficient murine and human cell lines that pharmacological PI3K inhibition increased AR protein thereby activating AR-related gene expression through a HER3 dependent mechanism (HER2 and Her3 promote AR activity and stability); similar effects were seen with AKT inhibition. They cross-validated this data in human samples indirectly demonstrating that a gene set enrichment score (GESA) of AR activity was significantly repressed in PTEN null human samples, as well as being associated with decreased HER2 expression [74,75]. Thereafter, they also demonstrated the inverse relationship with AR inhibition being associated with upregulated AKT signaling as a result of increased phosphorylation of AKT target genes such as GSK-alpha and PRAS40. The mechanism was determined to be through AR inhibition causing downregulation of the androgen dependent immunophilin FKBP5 that in turn is a chaperone for the AKT phosphatase PHLPP [8,76]. Finally, to confirm their finding of cross-regulation between the AR and PI3K pathways, they tested the effect of single pathway and combined pathway inhibition on PTEN deficient models. While single pathway inhibition with either enzalutamide or BEZ235 (a PI3K inhibitor) only had modest cytostatic effects, the combination of AR and PI3K pathway inhibition (in particular PI3K and/or mTORC 1/2) or PI3K inhibition and HER2/3 inhibition led to significant tumor reductions.

Utilizing a PTEN conditional murine prostate cancer model, Mulholland et al. demonstrated that PTEN loss suppresses AR transcriptional output and generally drives gene expression towards a castrate-like phenotype. To determine how PTEN loss causes suppression of AR transcriptional output, they used a doxycycline-dependent PTEN loss murine model. They found PTEN re-expression did not affect AR expression but did lead to reduced expression of EGR1 and c-JUN transcription factors, factors that are known to be up-regulated particularly in CRPC and to promote cancer growth in an androgen-depleted environment through direct interaction with and downregulation of the AR [77,78]. Through Network Component Analysis, they showed that PTEN re-expression was associated with reduced transcription factor activities (TFAs) of EGR1 and c-JUN followed by increased AR TFA. Reduced AR TFA seen in PTEN null models can be reversed by mTOR inhibition, suggesting involvement of the PI3K/AKT/mTOR pathway as seen by Carver et al. Additionally, Mulholland et al. found that downregulation of FKBP5/PHLPP by AR inhibition/loss may release the negative feedback on the AKT pathway to promote AKT-dependent, AR-independent cell growth. They showed more significant tumor regressions with dual pathway inhibition via Enzalutamide and rapamycin rather than single pathway inhibition in both PTEN null/AR+ prostate cancer cell lines and PTEN null mice.

Given the complexity of the AR and PI3K pathways, they likely interact at numerous levels. AR-induced PI3K stimulation may also occur through Src-mediated non-nuclear signaling, particularly in the context of ADT [79,80]. Androgen-bound AR can form a complex with Src to induce cell proliferation pathways. Aberrant Src signaling is present in prostate cancer cells. In low passage, androgen sensitive LNCaP prostate cancer cell lines, Src signaling is androgen-dependent. High passage cell lines however demonstrate constitutively activated AR/Src-induced proliferation in the absence of androgen [81]. AR and PI3K cross talk may occur through interactions of AR/Src and the p85α subunit of PI3K may trigger downstream pathway activation to promote cell survival in androgen-deplete conditions [10]. These interactions may be particularly important in patients treated with enzalutamide which prevents translocation of the AR into the nucleus promoting more cytosolic interactions which stimulate non-nuclear signaling.

Though Mulholland and Carver proposed somewhat different mechanisms, they independently demonstrated with both pharmacologic and genetic approaches PI3K/AKT activation via PTEN loss promotes prostate cancer growth in the absence of AR signaling; as a result, they hypothesize that strong suppression of AR-signaling with potent anti-androgen therapy may select for tumors with PI3K pathway activation and repressed AR activity leading to CRPC. They showed that dual pathway inhibition with androgen deprivation and a PI3K, AKT or mTOR inhibitor could lead to significant tumor regression as compared to single pathway inhibition.

Subsequent studies have supported the presence of AR-PI3K pathway interactions. Zhu et al. showed that conditional expression of human AR transgene in transgenic mice prostates not only induced malignancy but also resulted in decreased AKT activation in the tumor cells [82]. They further investigated the interaction between the PI3K/AKT and AR pathways in a series of in vitro and in vivo experiments [83] which confirmed a functional interaction between the pathways. They showed that depletion of androgens by various means results in increased expression of phosphorylated AKT and castration of conditional PTEN knockout mice increases AKT expression in prostate cancer cells. Furthermore, they demonstrated decreased endogenous AR expression in PTEN-null prostatic cells.

## 7. Therapeutic Implications

**Figure 1:**
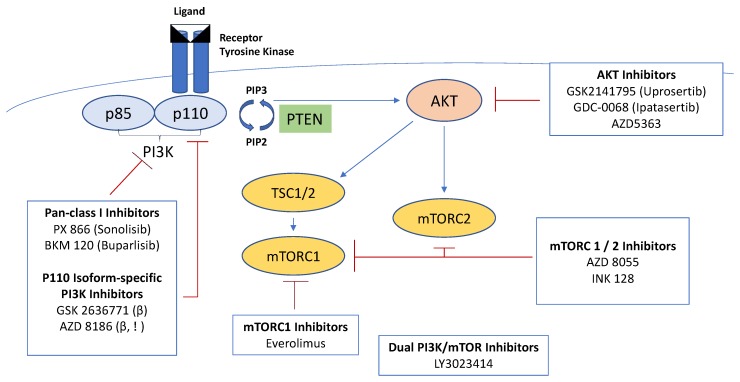
PI3K pathway targeting agents in development for the treatment of prostate cancer.

Recognition of the role the PI3K pathway plays in the development and propagation of cancer has led to the development of several PI3K inhibitors. Classes of drugs targeting the PI3K pathway and its downstream targets include pan-class I PI3K inhibitors, isoform-selective PI3K inhibitors, rapamycin analogues, active-site mTOR inhibitors, pan-PI3K/mTOR inhibitors and AKT inhibitors (Figure 1). Though some studies have yet to be reported, early studies in both pan-PI3K class I inhibitors and isoform-specific PI3K inhibitors have shown limited activity due to a combination of dose limiting toxicities, inadequate target inhibition and likely up-regulation of compensatory pathways [84,85,86]. For example, Hotte et al. presented data at ASCO 2013 on the use of PX-866, an irreversible pan-isoform inhibitor of class I PI3K, in men with mCRPC [87]. In this single-arm phase II study, 43 docetaxel-naïve men with mCRPC were treated with PX-866 with a primary endpoint of lack of progression at 12 weeks. Overall, PX-866 was well tolerated, but only 12 patients (28.4%) were progression-free at 12 weeks with one confirmed prostate-specific antigen (PSA) response. This agent did not meet the a priori benchmarks for further development as a single agent in unselected patients. Trials of monotherapy with AKT or mTOR inhibitors have also failed to progress. Burris et al., reported at ASCO 2011 the safety, pharmacokinetics and pharmacodynamics of the pan-AKT inhibitor GSK2141795 in nine prostate cancer patients of whom five were documented to have had PTEN loss [88]. In this cohort, seven patients had measurable responses, and six had stable disease with two having treatment durations in excess of 180 days; based on the phase I study results, development of this agent as monotherapy was not pursued, however. A recent systematic review of mTOR inhibition for mCRPC similarly found limited efficacy [89].

There are a number of explanations for the lack of efficacy seen in these trials of single pathway inhibition. Clinical correlation of the pre-clinical data on AR and PI3K pathway crosstalk was suggested in a phase I/II trial of everolimus, an mTOR inhibitor, in combination with gefitinib in patients with metastatic CRPC [90]. Rapid PSA rises occurred which often declined upon treatment discontinuation. In light of Carver and Mulholland’s work, these transient PSA rises may represent a surrogate marker of AR reactivation and AR-dependent transcription as a result of mTOR inhibition.

## 8. Combined Therapeutic Targeting of AR and PI3K Signaling

If the mutual inhibition of both pathways is required, and from the results above it seems that PI3K activation is not the sole route of standard androgen resistance, then the combination of AR targeting and PI3K targeting would appear to be intuitive. Studies currently underway in prostate cancer are particularly focused on using PI3K inhibitors to overcome castrate-resistance. Thus, PI3K inhibitors are largely being tested in combinations in patients who have progressed on either enzalutamide or abiraterone to test the hypothesis of emerging resistance to these agents via the PI3K pathway.

Hotte et al. presented the second part of their phase 2 study of PX-866 at ASCO GU 2015; 25 patients with progressive CRPC on abiraterone/prednisone were treated with a combination of PX-866 and continued abiraterone/prednisone [91]. Six patients (24%) were progression-free at 12 weeks, but no objective or PSA responses (PCWG2) were seen. Similarly, in another phase 2 study presented at the same meeting, PI3K inhibition with BKM120 with or without AR inhibition with enzalutamide failed to improve progression-free survival (PFS) in men with progressive CRPC on enzalutamide [92]. However, AKT inhibition with ipatasertib in combination with abiraterone improved radiographic PFS and overall survival (OS) in men with CRPC previously treated with docetaxel [93]. Unlike the two previous studies, only a small portion (23/253) of these patients had received treatment with a novel anti-androgen prior to enrolment. Two other phase 2 studies have been published exploring the combination of the mTOR inhibitor, everolimus and bicalutamide. Nakabayashi et al. reported a study of bicalutamide in combination with everolimus in which only two of 36 patients (6%) treated with bicalutamide in combination with everolimus achieved a PSA fall ≥50% [94]. Thirty-one (86%) of the men had been treated with bicalutamide previously. Chow et al. however, reported on 24 bicalutamide naïve men with CRPC treated with this combination based on a historic PSA response rate of 25% for bicalutamide alone in CRPC [95]. Though they achieved a PSA response (50% PSA fall) rate of 62.5%, this level of activity was abrogated by a high rate (54%) of grade 3 or 4 adverse events attributable to treatment.

These studies raise the question of whether earlier PI3K pathway inhibition, prior to development of castrate resistance or significant pre-treatment with androgen-targeted treatments, would be more efficacious. Some pre-clinical models have shown more durable responses to dual AR and PI3K pathway inhibition in castrate sensitive-cell lines as compared to castrate-resistant [96,97,98]. Another issue may be patient selection as most of the data are in unselected, heavily pre-treated patients.

## 9. Biomarkers for PI3K Inhibition

The prolonged responses seen in two of the patients presented by Burris et al. raise the question of whether patient selection may be another contributing factor to the lack of overall efficacy seen in many of these trials. Attempts to identify subpopulations that will yield maximum benefit from PI3K inhibitors are underway with testing for PIK3CA or AKT alterations and PTEN loss. Pre-clinical data indicate that tumors with PIK3CA mutations or PTEN loss are more sensitive to PIK3CA and AKT inhibition but the value of these markers in clinical practice is uncertain due to the complexity of the pathway and unknown effects of these agents on the tumor microenvironment [99]. Some PIK3CA mutations result in minimal activation of AKT as compared to PTEN loss suggesting that AKT inhibitors may be more efficacious in cancers with AKT alterations and PTEN loss [99,100]. One patient with mCRPC harboring a PIK3CA mutation treated with PX-866 on the phase I study achieved a prolonged clinical response to the PI3K inhibitor [84]. However, the predictive value of PIK3CA mutations has not been confirmed in other studies [85,101].

Most recently, de Bono, et al. presented data supporting PTEN loss as a predictor of response to treatment with ipatasertib in combination with abiraterone acetate in men with mCRPC [102]. PTEN expression was assessed by immunohistochemistry (IHC) in archival or fresh tumor samples and genomic loss was detected by fluorescence in situ hybridization (FISH) and next generation sequencing (NGS). Of the 253 patients randomized, PTEN IHC was evaluable in 165 with PTEN loss detected in 71 (41%). There was good concordance between IHC, FISH and NGS results. Median radiographic progression-free survival (rPFS) was 5.6 months vs. 7.5 months in the non-PTEN loss abiraterone plus placebo arm and abiraterone plus ipatasertib arms respectively. PTEN loss was associated with a shorter rPFS in the placebo plus abiraterone arm and a greater treatment effect in the 400 mg ipatasertib plus abiraterone arm (4.6 months and 11.5 months). Based on these results, this combination is planned to proceed to a phase III trial.

## 10. Current Clinical Trials of PI3K Pathway Inhibitors in Prostate Cancer

Table 1 details the different agents in development and the trials currently being conducted with these agents. Three of the five trials actively recruiting involve PI3K/AKT/mTOR agents in combination with anti-androgen therapy while another is examining combination with docetaxel. GSK2636771 is a p110β isoform-specific inhibitor with preliminary signs of activity in PTEN-deficient tumors [103]. AZD8186 inhibits both p110β and -δ isoforms and has demonstrated anti-tumor effects in vitro as monotherapy and in combination with docetaxel in prostate cancer models [104]. Interestingly, AZD8186 showed activity in both PTEN null and PTEN wildtype models. LY3023414 is a dual class I PI3K and mTOR inhibitor with phase I monotherapy data in advanced solid tumors [105]. AZD5363, on the other hand, is an inhibitor of AKT isoforms 1, 2 and 3 which has synergy with enzalutamide in preclinical models of enzalutamide-resistant prostate cancer and docetaxel in CRPC [106]. AZD5363 in combination with docetaxel is proceeding to a phase II study following determination of the recommended dose in the recently published ProCaid study in men with mCRPC [107]. In this study, 10 patients were treated, of whom seven (70%) had a >50% reduction of PSA from baseline to 12 weeks. The most common toxicities were rash and diarrhea with self-limiting hyperglycemia seen in all patients.

## 11. Conclusions

The androgen receptor and PI3K pathways are the two most commonly deregulated pathways in prostate cancer. There is evidence that PI3K signaling is involved in the evolution to castrate-resistant disease, a form of prostate cancer that remains lethal despite recent advances. This understanding has led to the development of several drugs targeting the PI3K pathway and its downstream targets but, unfortunately, early results overall have been disappointing. Adding complexity to early trials is the issue of interpreting a rising PSA, the most commonly measured marker of response in prostate cancer, in the context of potential activation of AR transcription with resultant PSA rises following PI3K pathway inhibition. Pre-clinical data supporting combined pathway inhibition coupled with the lack of substantial single-agent activity have prompted studies of PI3K pathway inhibition in combination with androgen pathway inhibition and/or additional downstream AKT/mTOR inhibition; the results continue to be mixed with efficacy often compromised by toxicity. There is a suggestion that earlier treatment with these agents, to prevent rather than overcome castrate-resistance, may be a useful strategy.

Ongoing studies to address the optimal timing, sequence and combinations of these treatments in addition to potential predictive biomarkers are underway. Given the reciprocal activation of p110α upon p110β inhibition in PTEN null tumors, it will be interesting to see the outcomes with the isoform-specific PI3K inhibitors in combination with enzalutamide. It is unclear whether the preferred agent should target multiple nodes of the pathway, such as with LY3023414, or induce pan-isoform inhibition of a single node, such as with AZD 5363. Despite their promise, it is yet to be seen whether these strategies can successfully overcome endocrine resistance to yield a significant improvement in outcomes for patients.

## Figures and Tables

**Table 1:** PI3K and AKT inhibitor agents in clinical development.

Agent	Pharmaceutical Company	Sponsor	Trial	Endpoint	Patient Population	Status	Biomarkers
BKM120 (PI3K)	Novartis	Duke University	Phase II, BKM120 in mCRPC (NCT01385293)	PFS	Post chemo; prior sipuleucel-T, abiraterone (Abi), or enzalutamide (enza) allowed. *n* = 66	Study accrued, results awaited	Circulating tumor cells (CTCs), Tissue PI3K signature, PTEN status, PI3K activation, PSA levels
		University of California	Phase II, neoadjuvant BKM120 for high-risk prostate cancer pre radical prostatectomy (RP) (NCT01695473)	PI3K inhibition in tumor measured by IHC	Candidates for RP; high risk defined by trial Target *n* = 24	Study accrued, results awaited	IHC for phosphorylation of: S6, 4EBP1, or AKT
GSK2636771 (PI3K)	GlaxoSmithKline	GSK	Phase I, GSK2636771 in combination with Enza for mCRPC (NCT02215096)	Safety and tolerability	PTEN deficient tumors post progression on Enza *n* = 44	Recruiting	PTEN status PSA levels
AZD8186 (PI3K)	AstraZeneca	AZ	Phase I, AZD8186 +/− Abi or AZD2014 in TNBC/NSCLC or CRPC or known PTEN-deficient/PI3 mutated disease (NCT01884285)	Safety and tolerability	mCRPC (Total) Target *n* = 180	Recruiting	PSA levels
LY3023414 (PI3K + mTOR)	Eli Lilly	Eli Lilly	Phase II Study of Enzalutamide +/− LY3023414 in mCRPC (NCT02407054)	PFS	mCRPC post progression on Abi; no prior chemo in castrate-refractory setting, immunotherapy, or Ra223 Target *n* = 144	Recruiting	PSA levels
AZD5363 (AKT)	AstraZenica	Institute of Cancer Research, UK	Phase I/II, Enza +/− AZD5363 in mCRPC	Phase II: Best overall tumor response	mCRPC with tissue for PTEN testing Target *n* = 136	Recruiting	PTEN PSA levels
		University Hospital Southampton NHS Foundation Trust	Randomised Phase II, Docetaxel +/− AZD 5363 in mCRPC	PFS	Chemotherapy-naïve mCRPC	Recruiting	PSA levels
MK2206 (AKT)	Merck	National Cancer Institute	Phase II, bicalutamide +/− MK2206 in men with HSPC	Proportion undetectable PSA	Biochemically relapsed hormone-sensitive PC following definitive treatment *n* = 104	Study accrued, results awaited	PSA levels
